# Effects of Glucagon-Like Peptide-1 Receptor Agonists and Sodium-Glucose Cotransporter 2 Inhibitors on Intima-Media Thickness: Systematic Review and Meta-Analysis

**DOI:** 10.1155/2024/3212795

**Published:** 2024-03-18

**Authors:** Abolfazl Akbari, Shiva Hadizadeh, Leida Heidary

**Affiliations:** ^1^Student Research Committee, Faculty of Medicine, Mashhad University of Medical Sciences, Mashhad, Iran; ^2^Research Center for Advanced Technologies in Cardiovascular Medicine, Cardiovascular Research Institute, Tehran University of Medical Sciences, Tehran, Iran; ^3^Women Reproductive Health Research Center, Tabriz University of Medical Sciences, Tabriz, Iran; ^4^Laboratory of Medical Genetics, ART and Stem Cell Research Centre (ACECR), Tabriz, Iran; ^5^Nahal Infertility Center, Tabriz, Iran

## Abstract

**Background:**

Beyond glycemic control, glucagon-like peptide-1 receptor agonists (GLP-1 RAs) and sodium-glucose cotransporter 2 inhibitors (SGLT2is) have been proposed to reduce the risk of cardiovascular events. The aim of the present systematic review and meta-analysis is to demonstrate the effects of GLP-1 RA and SGLT2is on intima-media thickness (IMT).

**Methods:**

PubMed, EMBASE, Web of Science, SCOPUS, and Google Scholar databases were searched from inception to September 9, 2023. All interventional and observational studies that provided data on the effects of GLP-1 RAs or SGLT2is on IMT were included. Critical appraisal was performed using the Joanna Briggs Institute checklists. IMT changes (preintervention and postintervention) were pooled and meta-analyzed using a random-effects model. Subgroup analyses were based on type of medication (GLP-1 RA: liraglutide and exenatide; SGLT2i: empagliflozin, ipragliflozin, tofogliflozin, and dapagliflozin), randomized clinical trials (RCTs), and diabetic patients.

**Results:**

The literature search yielded 708 related articles after duplicates were removed. Eighteen studies examined the effects of GLP-1 RA, and eleven examined the effects of SGLT2i. GLP-1 RA and SGLT2i significantly decreased IMT (MD = −0.123, 95% CI (-0.170, -0.076), *P* < 0.0001, *I*^2^ = 98% and MD = −0.048, 95% CI (-0.092, -0.004), *P* = 0.031, *I*^2^ = 95%, respectively). Metaregression showed that IMT change correlated with baseline IMT, whereas it did not correlate with gender, duration of diabetes, and duration of treatment.

**Conclusions:**

Treatment with GLP-1 RA and SGLT2i can lower IMT in diabetic patients, and GLP-1 RA may be more effective than SGLT2i.

## 1. Introduction

Glucagon-like peptide-1 (GLP-1) receptor agonists (RAs) and sodium/glucose cotransporter 2 inhibitors (SGLT2is) have been introduced for the treatment of type 2 diabetes mellitus (T2DM). Their promising results in glycemic control and weight loss, as well as their low risk of hypoglycemia, less adverse events, and favourable renocardiovascular effects have made them desirable therapies for the treatment of T2DM and its concomitant diseases and complications [1]. In addition to their putative target, numerous molecular targets for GLP-1s have been identified, justifying their potential for broader medical applications, including autophagy, oxidative stress, platelet function, lipid metabolism, and inflammation [2–9]. GLP-1 RA causes an increase in insulin secretion and a decrease in glucagon levels in response to glucose and delays gastric emptying, thereby suppressing postprandial hyperglycemia and appetite, resulting in a decrease in total energy intake and body weight [10]. SGLT2is act independently of insulin; they block renal glucose reabsorption mediated by SGLT2 expressed along proximal tubules and cause glucosuria [7].

Carotid intima-media thickness (IMT) is a quick and noninvasive ultrasound marker that indicates the thickness of the two innermost layers of the carotid artery. It is a risk stratification tool used as a surrogate marker for atherosclerosis in numerous studies to assess the risk of cardiovascular events [11–13]. We are interested in comparing the effects of GLP-1 RA and SGLT2i therapies on IMT, which may reflect the cardioprotective effects of these drugs. A direct comparison of the cardioprotective benefits of two second-line therapies in T2DM could help us find a better strategy for glycemic control. However, no systematic comparison has been performed for GLP-1 RA or SGLT2i therapies in terms of their effect on IMT. Therefore, we performed a comprehensive systematic review and meta-analyses to determine the effects of GLP-1 RA and SGLT2i drugs on IMT.

## 2. Methods

This systematic review is in accordance with the Preferred Reporting Items for Systematic Reviews and Meta-Analyses (PRISMA) 2020 statement [14]. The study protocol was registered with the International Prospective Registry of Systematic Reviews (PROSPERO).

### 2.1. Data Sources and Searching Strategy

To identify potentially relevant studies, searches were conducted in the following four databases (since inception to September 9, 2023): PubMed, EMBASE, Web of Science, and SCOPUS, with two reviewers (A.A. and S.H.) working independently and in parallel. Citations of all included studies and relevant published papers were reviewed by hand search. “Google Scholar” was also searched to find potentially relevant articles. Studies were found by searching for three main terms and their synonyms, including “IM,” “SGLT2i,” and “GLP-1 RA.” The complete search strategy for each term is shown in Table S1. The search was not limited by time, type of article, or language. We used reference management software (EndNote X8) to import references, remove duplicates, and review the literature.

### 2.2. Selection Criteria

Inclusive criteria for this systematic review were studies that investigated IMT in groups of patients treated with GLP-1 RA or SGLT2i. Eligible studies that met the following criteria were included in the meta-analysis: (1) the studies reported mean IMT at baseline and final or mean change in IMT after GLP-1 RA or SGLT2i therapy, and (2) the follow-up period was at least 2 weeks. Two authors (A.A. and S.H.), working independently and in parallel, reviewed the abstract and included the paper reporting the effects of GLP-1 RA or SGLT2i on IMT. Subsequently, A.A. and S.H. independently assessed the full text of the papers and made the final decision. Disagreements in study selection were adjudicated by a third reviewer.

### 2.3. Quality Assessment

Two authors (L.H. and S.H.) independently assessed the quality of studies using the JBI checklists [15]. The JBI checklist assessed bias in selection, measurement, and analysis. If there were disagreements, they were resolved by discussion or referral to another investigator to achieve consensus. The checklist questions were answered “yes,” “no,” “unclear,” or “not applicable.” For each “yes” answer, 1 point is awarded, and after adding the points, the final score is calculated.

### 2.4. Data Extraction

The two investigators (A.A. and S.H.) independently extracted the following data: first name, year in which studies were conducted (if no data were provided, the year of study publication was considered), groups, dosage, population, size, gender, age, location, study design, follow-up, IMT at baseline, IMT at end, and disease duration.

### 2.5. Publication Bias and Statistical Analysis

Publication bias was examined using funnel plots, Egger's test, and Duval and Tweedie's trim and fill test. Pre- and postintervention IMT values were recorded to calculate the mean difference (MD) and 95% confidence interval (CI). Subgroup analyses were performed based on drug classes, and sensitivity analyses were performed based on effect models (random to fixed or vice versa), RTCs, T2DM patients, and *R* values (0.3, 0.5, and 0.8). The Cochrane *Q* statistic was used to assess heterogeneity, and if it was less than 0.05, a random-effects model was used for analysis. Metaregression was performed to determine the correlation between IMT changes and disease duration, gender, follow-up period, and baseline IMT. A *P* value of less than 0.05 was considered statistically significant for the outcome and heterogeneity analyses. Data analysis was performed using Comprehensive Meta-Analysis software (CMA) V.3.

## 3. Results

**Figure 1:**
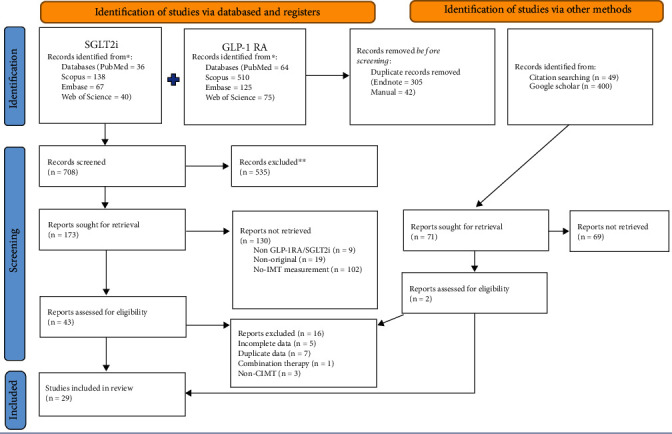
PRISMA flow diagram of the systematic review process.

The literature search yielded 708 related articles after duplicates were removed. Eighteen studies examined the effects of GLP-1 RA [11–13, 16–31], and eleven examined the effects of SGLT2i on IMT [32–42]. Studies that did not provide IMT results [43–49], duplicate data [50–56], combination therapies without apparent GLP-1 RA effects [57], or assessed IMT of arteries other than the carotid artery were excluded [58–60]. The study selection process is shown in Figure 1.

### 3.1. Characteristics of the Included Studies

Three different GLP-1 RA drugs were investigated in the included studies: liraglutide [11, 12, 16, 18, 23–29], semaglutide [21], and exenatide [19, 22, 30, 31]. Also, five different SGLT2i drugs were studied, including empagliflozin [32, 36, 38, 42], ipragliflozin [33, 39, 41], tofogliflozin [34, 40], dapagliflozin [35–37, 42], and luseogliflozin [40]. The range of intervention periods for GLP-1 RA trials ranged from 4 months [12, 13, 21, 26] to 3 years [29] and for SGLT2i trials was from 2 weeks [38] to 3.6 years [36]. All SGLT2i studies [32–42] and fifteen GLP-1 RA studies included T2DM patients [11, 12, 17, 19–27, 29–31]. Italy (*n* = 7) was the country with the largest number of published articles for GLP-1 RA and Japan for SGLT2i (*n* = 5). Characteristics of the evaluated studies are presented in Table 1.

### 3.2. GLP-1 RA

**Figure 2:**
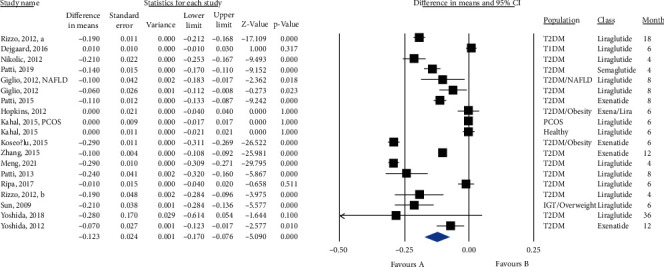
Forest plot displaying pre-post difference and 95% confidence interval for the impact of GLP-1 RA on IMT.

**Figure 3:**
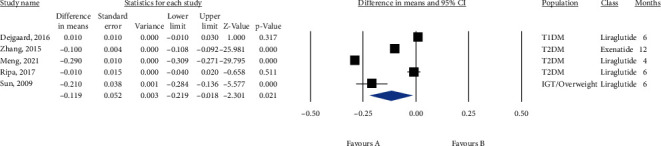
Forest plot displaying pre-post difference and 95% confidence interval for the impact of GLP-1 RA on IMT based on randomized clinical trials.

**Figure 4:**
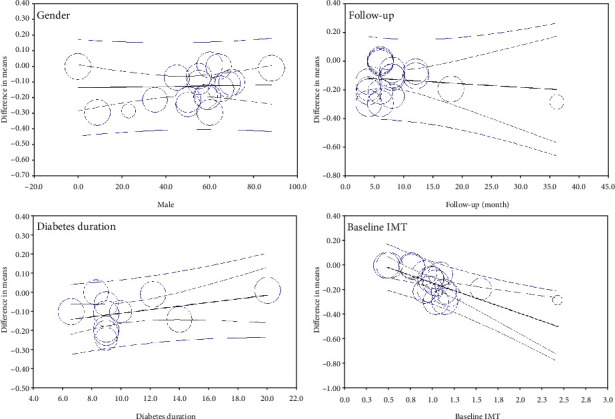
Meta-regression plots of the association between IMT with gender, follow-up, duration of diabetes, and baseline IMT for GLP-1 RA studies.

Nineteen GLP-1 RA-treated groups with a total population of 790 subjects were included in the meta-analysis.  Figure 2 shows that GLP-1 RA significantly reduced IMT (MD = −0.123, 95% CI (-0.170, -0.076), *P* < 0.0001, *I*^2^ = 98%). A sensitivity analysis on studies that included only T2DM patients showed a higher potential of GLP-1 RA to reduce IMT (MD = −0.145, 95% CI (-0.196, -0.094), *P* < 0.0001, *I*^2^ = 98%) (Figure S1). In addition, a sensitivity analysis based on 5 RCTs reached the same conclusion (MD = −0.119, 95% CI (-0.219, -0.018), *P* = 0.021, *I*^2^ = 99%) (Figure 3). A subgroup analysis on liraglutide and exenatide trials significantly reduced IMT (liraglutide: MD = −0.127, 95% CI (-0.201, -0.054), *P* = 0.001, *I*^2^ = 99%; exenatide: MD = −0.144, 95% CI (-0.240, -0.047), *P* = 0.003, *I*^2^ = 99%) (Figure S2). Metaregression showed that IMT change was significantly correlated with baseline IMT (coefficient = −0.246, *P* = 0.0001) but not significantly correlated with duration of treatment, duration of diabetes, and gender (coefficient = −0.003, *P* = 0.635; coefficient = 0.009, *P* = 0.108; and coefficient = 0.001, *P* = 0.849, respectively) (Figure 4).

**Figure 5:**
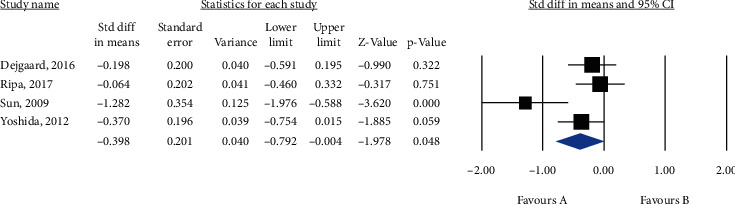
Forest plot displaying mean difference and 95% confidence interval for the impact of GLP-1 RA compared to control groups on IMT.

Meta-analysis of 4 studies (*n* = 343) showed a significant reduction in the GLP-1 RA group compared with the placebo/control group (MD = −0.398, 95% CI (-0.792, -0.004), *P* = 0.048, *I*^2^ = 68%) (Figure 5).

### 3.3. SGLT2i

**Figure 6:**
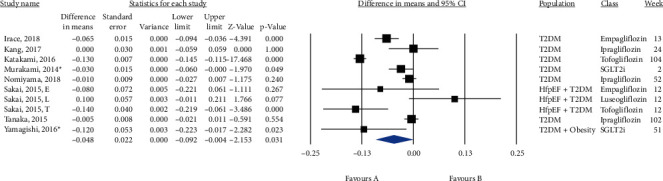
Forest plot displaying pre-post difference and 95% confidence interval for the impact of SGLT2i on IMT.

**Figure 7:**
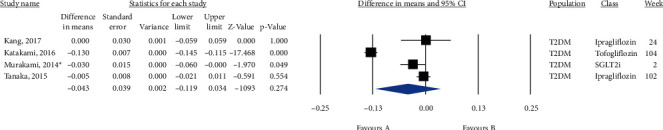
Forest plot displaying pre-post difference and 95% confidence interval for the impact of SGLT2i on IMT based on randomized clinical trials.

**Figure 8:**
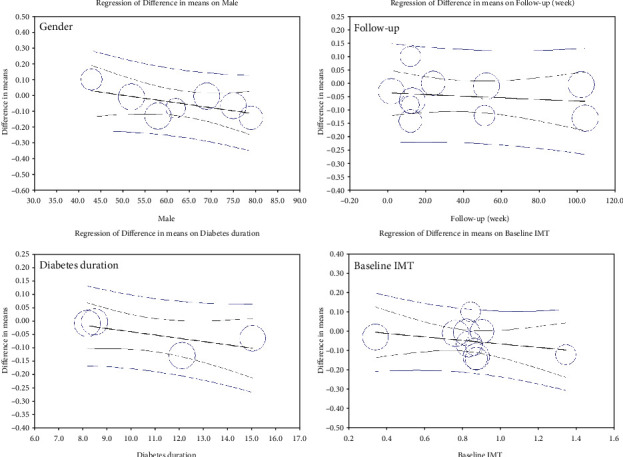
Metaregression plots of the association between IMT with gender, follow-up, duration of diabetes, and baseline IMT for SGLT2i studies.

Ten groups treated with SGLT2i with a total population of 879 subjects were included in the meta-analysis.  Figure 6 shows that SGLT2i could significantly reduce IMT (MD = −0.048, 95% CI (-0.092, -0.004), *P* = 0.031, *I*^2^ = 95%). In addition, a sensitivity analysis based on 4 RCTs reached the same conclusion (MD = −0.043, 95% CI (-0.119, 0.034), *P* = 0.274, *I*^2^ = 98%) (Figure 7), but the sensitivity analysis based on the change from random to fixed effects showed a significant reduction in IMT (MD = −0.067, 95% CI (-0.077, -0.057), *P* = 0.0001). A subgroup analysis on empagliflozin and tofogliflozin trials significantly reduced IMT (empagliflozin: MD = −0.066, 95% CI (-0.094, -0.037), *P* < 0.0001, *I*^2^ = 0%; tofogliflozin: MD = −0.130, 95% CI (-0.145, -0.116), *P* < 0.0001, *I*^2^ = 0%), whereas ipragliflozin failed to reduce IMT (MD = −0.007, 95% CI (-0.019, 0.004), *P* = 0.222, *I*^2^ = 0%) (Figure S3). Metaregression showed that IMT change was not significantly correlated with baseline IMT, treatment duration, diabetes duration, and gender (coefficient = −0.092, *P* = 0.363; coefficient = −0.001, *P* = 0.623; coefficient = −0.012, *P* = 0.178, and coefficient = −0.004, *P* = 0.171, respectively) (Figure 8).

All comparisons were repeated by changing the *R* value to 0.3, 0.5, or 0.8, but no differences were found. A sensitivity analysis in which the random-effects analysis was replaced by a fixed-effects analysis also confirmed the results, except as noted in the manuscript.

### 3.4. Quality Assessment and Publication Bias

**Figure 9:**
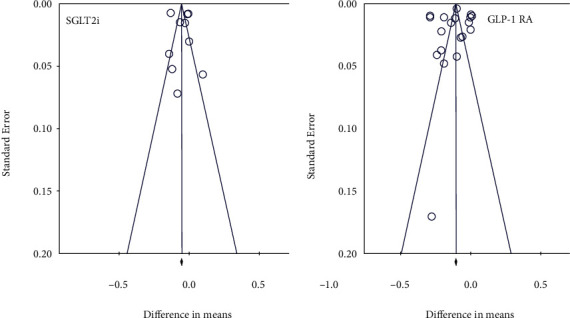
Funnel plot displaying the impact of GLP-1 RA and SGLT2i on IMT.

Quality assessment using the JBI checklist and final scores for cohort studies, cross-sectional studies, RCTs, and nonrandomized clinical trials are described in detail in Table S2. All funnel plots of all analyses are shown in the figures.  Figure 9 shows the funnel plot of the pre-post comparison of GLP-1 RA and SGLT2i treatment, and Figure S4 shows the funnel plots of the sensitivity analysis. In addition, the results of the Egger test and the trim-and-fill method of Duval and Tweedie, which indicate no significant publication bias, are shown in Table 2 (Table 2).

## 4. Discussion

T2DM is associated with a high prevalence of cardiovascular risk, and pharmacotherapies have been introduced to reduce the risk of atherosclerosis in various ways, including glycemic control, lipid balance, uric acid lowering, and blood pressure control [61]. Our meta-analysis showed a significant reduction in IMT, a surrogate atherosclerosis marker, after GLP-1 RA or SGLT2i therapy; however, it appears that GLP-1 RA is more effective in reducing IMT. Similarly, a recent meta-analysis of RCTs showed that GLP-1 RAs were effective in preventing serious adverse cardiovascular events in T2DM patients with obesity (relative risk = 0.88, 95% CI (0.81, 0.96)), whereas SGLT2i marginally prevented serious adverse cardiovascular events (relative risk = 0.91, 95% CI (0.83, 1.00)) [62]. In contrast to a recent review showing cardiovascular benefits for liraglutide and semaglutide but not for exenatide, we demonstrated that exenatide can also reduce IMT [63]. It appears that the effects of GLP-1 RA are not class-dependent, whereas the effects of SGLT2i are.

Consistent with our findings, previous studies reported that GLP-1 RA was effective in reducing major adverse events associated with cardiac events regardless of gender. In contrast, in terms of reducing major adverse events associated with cardiac events, SGLT2i was effective in men but not in women [64]. This inconsistency may be due to different outcome measures. Metaregression analysis showed that the effects of GLP-1 RA and SGLT2i persisted with long-term treatment, suggesting that these drugs do not induce tolerance. In contrast to a previous study claiming that the duration of T2DM might influence efficacy, the metaregression showed no significant correlation between the duration of diabetes and change in IMT [65]. However, the metaregression showed that higher baseline IMT leads to greater IMT reduction. A study by Kahal et al. showed that GLP-1 RA was not significantly effective in patients with polycystic ovary syndrome whose baseline IMT was lower than that of T2DM patients [18]. In these cases, confounding factors and heterogeneity may affect the results, so well-designed RCTs are warranted.

A meta-analysis by Song et al. evaluated the efficacy of GLP-1-based therapies and concluded that IMT was not significantly reduced. Insufficient studies, heterogeneity, and pooling other GLP-1-based therapy other than GLP-1 RA including dipeptidyl peptidase-4 inhibitors may lead to different results compared with our findings [66]. They also showed that brain natriuretic peptide, a marker of atherosclerosis, decreased significantly with GLP-1-based therapies. Furthermore, in a prospective study of elderly people in Sweden, it was observed that higher serum GLP-1 levels correlated with lower IMT [67].

Prior studies suggest that liraglutide can regulate the NLRP3 inflammasome and NF-*κ*B signaling pathway, which causes the inflammatory state [28, 68, 69]. It has also been shown that GLP-1 RA protects cardiomyocytes from IL 1*β*-induced metabolic dysfunction and mitochondrial dysfunction [70]. Previous studies have shown that GLP-1 RA therapy lowers both systolic and diastolic blood pressure [71], improves endothelial dysfunction [72, 73], and reduces macrophage foam cell formation and atherosclerosis [74]. Qu and Qu reviewed evidence from epidemiological and human studies that low-density lipoprotein cholesterol (LDL-C) is an important regulator in the development of atherosclerosis [75]. A previous meta-analysis by Zhao et al. showed a significant reduction in LDL-C following GLP-1 RA therapy [76], whereas Sánchez-García et al. did not achieve a significant reduction after SGLT2is [77]. Another mechanism described for GLP-1 RA agonists is that these drugs increase antioxidant enzymes (superoxide dismutase and glutathione reductase) and decrease reactive oxygen species and malondialdehyde levels [78]. In vivo studies have shown that GLP-1 RA reduces atherosclerosis by suppressing endoplasmic reticulum stress, macrophage apoptosis, and microvesicle production [79]. Hyperglycemia has also been shown to lead to a decrease in endothelial nitric oxide function via a decrease in synthesis and an increase in degradation and to play a role in endothelial dysfunction, with liraglutide effectively restoring endothelial nitric oxide synthase activity in the diabetic mouse model [80, 81]. The same mechanism involving amelioration of inflammation, insulin resistance, endothelial dysfunction, dislipidemia, hyperglycemia, and oxidative state has been proposed for SGLT2is [82–86]. Previous studies have demonstrated the importance of AT1R/NADPH oxidase/SGLT1 and 2 signaling pathways in promoting atherosclerosis [87–89]. They showed that atherosclerotic plaques have higher SGLT2 expression [87–89]. A recent meta-analysis summarizing data from 9 RCTs and 2 cohorts concluded that SGLT2i improves flow-mediated dilation but not pulse wave velocity [90].

The paucity of high-quality randomized clinical trials in this systematic review is one of the major limitations of the current study. Lacking sufficient studies, we could not evaluate the effects of different classes, different doses, or patient characteristics of GLP-1 RA or SGLT2i on IMT. Also, there were insufficient studies to compare SGLT2i with control groups. There was too much heterogeneity, which could be due to different inclusion criteria, different types and dosages of GLP-1 RA or SGLT2i, follow-up time, and study design. Despite these aforementioned biases, sensitivity analyses yielded nearly consistent results.

In conclusion, GLP-1 RA and SGLT2i may reduce IMT. Among the different GLP-1 RAs, liraglutide was the most studied and had a significant effect on IMT reduction. In addition, GLP-1 RAs might be more effective than SGLT2is in lowering IMT.

## Figures and Tables

**Table 1:** Characteristics of included studies.

Study, year	Groups, dosage (per day)	Size, population	Male	Age (years)	Location	Study design	Follow-up	Baseline CIMT (mm)	Final CIMT (mm)	Disease duration (years)
*GLP-1 RA*										
Rizzo et al., 2012 [26]	Liraglutide 1.2 mg add on metformin 1500 mg	121 T2DM	59%	62 ± 9	Italy	Uncontrolled clinical trial	18 months	0.97 ± 0.18	0.78 ± 0.20	9 ± 8
Dejgaard et al., 2016 [16]	Liraglutide 1.8 mg Placebo	50 T1DM 50 T1DM	60% 70%	47 ± 13 49 ± 12	Denmark	Randomized, double-blinded clinical trial	24 weeks	ND	0.01 (-0.01, 0.03)/*p* = 0.361	20 ± 12 25 ± 12
Nikolic, 2021 [13]	Liraglutide 1.2 mg add on metformin	62 T2DM	50%	61 ± 9	Italy	Uncontrolled clinical trial	4 months	1.13 ± 0.29	0.92 ± 0.24	9 ± 8
Patti et al., 2019 [22]	Semaglutide 0.50 mg/week add on routine treatment	40 T2DM	65%	66 ± 10	Italy	Retrospective	4 months	1.04 ± 0.16	0.90 ± 0.14	14 ± 10
Giglio, 2014 [11]	Liraglutide add on metformin 1500-3000 mg	29 T2DM + NAFLD 29 T2DM	55% 55%	61 ± 10 61 ± 8	Italy	Clinical trial	8 months	1.00 ± 0.30 0.91 ± 0.23	0.90 ± 0.10 0.85 ± 0.15	10 ± 9 9 ± 8
Patti, 2023 [21]	Exenatide LAR 2 mg/week add on metformin 1500-3000 mg	60 T2DM	68%	60 ± 10	Italy	Uncontrolled clinical trial	8 months	0.98 ± 0.14	0.87 ± 0.15	9 ± 8
Hopkins, 2013 [17]	Exenatide 20 *μ*g Liraglutide 1.2 mg	9 T2DM + obesity 2 T2DM + obesity	63.6%	55 ± 8	United Kingdom	Uncontrolled clinical trial	6 months	0.76 ± 0.07	0.76 ± 0.11	8.3 ± 4.7
Kahal et al., 2015 [18]	Liraglutide 1.8 mg	13 PCOS 12 controls	0%	33.9 ± 6.7 33.5 ± 7.1	United Kingdom	Clinical trial	6 months	0.51 ± 0.05 0.48 ± 0.06	0.51 ± 0.05 0.48 ± 0.06	ND
Köseoğlu, 2021 [19]	Exenatide 20 *μ*g	45 T2DM + obesity	8.8%	47.91 ± 7.30	Turkey	Uncontrolled clinical trial	6 months	1.04 ± 0.11	0.75 ± 0.12	ND
Zhang, 2022 [31]	Exenatide Insulin	27 T2DM 32 T2DM	70.0% 43.7%	58.85 ± 12.54 58.03 ± 13.32	China	Randomized, open-label clinical trial	52 weeks	ND	-0.1 0.02 (change from baseline)	6.59 ± 5.32 7.81 ± 6.02
Luna-Marco et al., 2023 [20]	GLP-1 RA Non-GLP-1 RA Control	59 T2DM 196 T2DM 175 Control	63% 55% 54%	56.5 ± 9.9 59.9 ± 10.5 54.9 ± 13.5	Spain	Cross-sectional	ND	ND	0.630 ± 0.742 0.750 ± 0.238 0.516 ± 0.070	13.8 ± 8.7 10.4 ± 8.1
Meng, 2023† [12]	Liraglutide Metformin and sulfonylurea	38 T2DM 40 T2DM	60.5% 45%	56 ± 11 59 ± 7	China	Randomized clinical trial	16 weeks	1.14 ± 0.10 1.13 ± 0.13	0.85 ± 0.08 1.05 ± 0.10	0.5-16.0 1.0-16.0
Patti et al., 2013 [23]	Liraglutide 1.2 mg add on metformin	64 T2DM	50%	63 ± 8	Italy	Uncontrolled clinical trial	8 months	1.19 ± 0.47	0.95 ± 0.21	9 ± 8
Ripa, 2021 [24]	Liraglutide 1.8 mg Placebo	50 T2DM 48 T2DM	88.2% 80.4%	65.9 ± 8.6 66.9 ± 7.8	Denmark	Randomized, double-blinded clinical trial	26 weeks	0.77 ± 0.17 0.75 ± 0.14	0.76 ± 0.17 0.75 ± 0.14	12.2 ± 3.2 11.3 ± 3.4
Rizzo et al., 2012 [26]	Liraglutide 1.2 mg add on metformin 1500 mg	33 T2DM	58%	59 ± 9	Italy	Uncontrolled clinical trial	4 months	1.55 ± 0.45	1.36 ± 0.31	ND
Sun, 2023 [28]	Liraglutide 1.2 mg Lifestyle interventions	17 IGT + overweight 22 IGT + overweight	35.3% 31.9%	44.92 ± 14.69 48.91 ± 10.12	China	Randomized, double-blinded clinical trial	6 months	0.91 ± 0.25 0.91 ± 0.23	0.70 ± 0.16 0.92 ± 0.18	ND
Yoshida et al., 2018 [29]^∗^‡	Liraglutide 0.9 mg Linagliptin	34 T2DM	23.5%	75.7 ± 7.8	Japan	Clinical trial	3 years	2.42 ± 1.65 2.25 ± 1.19	2.14 ± 1.40 2.19 ± 1.01	ND
Yoshida et al., 2012 [30]^∗^	Exenatide 20 *μ*g add on routine treatment Routine treatment	56 T2DM 50 T2DM	44.6% 44.6%	63.8 ± 11.0 63.8 ± 11.0	Japan	Clinical trial	12 months	1.09 ± 0.33 1.08 ± 0.27	1.02 ± 0.31 1.13 ± 0.34	ND
*SGLT2i*										
Irace et al., 2018 [32]	Empagliflozin Incretin-based therapy	40 T2DM 30 T2DM	75% 80%	58 ± 9 60 ± 7	Italy	Prospective cohort	3 months	0.831 ± 0.156 0.890 ± 0.146	0.766 ± 0.127 0.841 ± 0.109	15 ± 9 17 ± 10
Kang, 2023 [33]	Ipragliflozin 50 mg Sitagliptin 100 mg	70 T2DM 70 T2DM	ND	ND	South Korea	Randomized, open-label clinical trial	24 weeks	0.900 ± 0.420 0.830 ± 0.230	0.900 ± 0.360 0.840 ± 0.250	ND
Katakami, 2022 [34]	Tofogliflozin 20 mg Conventional therapy	169 T2DM 171 T2DM	58.3% 58.0%	61.4 ± 9.3 60.8 ± 9.7	Multicenter (Japan)	Randomized, open-label clinical trial	104 weeks	0.870 ± 0.160 0.860 ± 0.150	0.740 ± 0.140 0.720 ± 0.130	12.1 ± 8.4 12.4 ± 8.2
Korzh et al., 2020 [35]^∗^	Dapagliflozin 10 mg	35 T2DM	ND	ND	Ukraine	Clinical trial	12 weeks	ND	Decreased significantly from baseline	ND
Kourtidou, 2023 [36]	Empagliflozin/dapagliflozin Standard care	15 T2DM 25 T2DM	73.3% 68%	68.9 ± 7.3 73.2 ± 9.6	Greece	Cross-sectional	3.6 ± 1.2 years	ND	0.7 ± 0.2 0.9 ± 0.2 (no significant difference)	12.6 ± 9.1 13.3 ± 7.1
Lamaida, 2022^∗^ [37]	Dapagliflozin Standard care	20 T2DM 20 T2DM	ND	55 ± 10 50 ± 10	Italy	Clinical trial	2.0 years	ND	Decreased significantly from baseline	ND
Murakami and Mizuno, 2014 [38]^∗^	SGLT2i Standard care	10 T2DM 10 T2DM	ND	ND	ND	Randomized clinical trial	2 weeks	0.340 ± 0.080 0.350 ± 0.080	0.310 ± 0.060 0.360 ± 0.050	ND
Nomiyama et al., 2018 [39]	Ipragliflozin 50 mg	134 T2DM	52%	53.9 ± 10.5	Japan	Clinical trial	52 weeks	0.760 ± 0.160	0.750 ± 0.150	8.2 ± 7.9
Sakai, 2019 [40]	Empagliflozin 10-25 mg Luseogliflozin 2.5-5 mg Tofogliflozin 20 mg	59 HfpEF + T2DM 63 HfpEF + T2DM 62 HfpEF + T2DM	61.5% 42.9% 78.6%	62.0 ± 9.4 70.3 ± 11.4 66.0 ± 9.8	Japan	Clinical trial	12 weeks	0.860 ± 0.700 0.840 ± 0.700 0.870 ± 0.500	0.780 ± 0.200 0.940 ± 0.400 0.730 ± 0.300	ND
Tanaka, 2023 [41]	Ipragliflozin 50 mg Standard care	241 T2DM 215 T2DM	69.4% 67.2%	67 (60, 72) 68 (60, 73)	Multicenter (Japan)	Randomized, open-label clinical trial	24 months	0.8200 ± 0.037 0.8400 ± 0.037	0.815 ± 0.034 0.836 ± 0.039	9.1 ± 6.8 8.1 ± 6.9
Yamagishi et al., 2016 [42] ^∗^	SGLT2i	31 T2DM + obesity	ND	53	Japan	Clinical trial	12 months	1.340 ± 0.480	1.220 ± 0.437	ND

^∗^Conference papers. †Chinese language. ‡Max MIT. Abbreviations: HfpEF: heart failure with preserved ejection fraction; T2DM: type 2 diabetes mellitus; PCOS: polycystic ovary syndrome; NAFLD: nonalcoholic fatty liver disease; IGT: impaired glucose tolerance; ND: not determined.

**Table 2:** Publication bias evaluation by Egger's regression test and Duval and Tweedie trim and fill test.

Finding/distribution pattern	Egger's test		Trim and fill method		
	Egger's intercept	*P* value	Number of trimmed studies	Point estimate after trim	Change after trim
Baseline-final GLP-1 RA	-1.191	0.706	1	-0.113	0.010
Baseline-final GLP-1 RA (RCTs)	-1.172	0.917	0	-0.119	0.000
Baseline-final GLP-1 RA (T2DM)	-1.685	0.565	0	-0.196	0.000
Baseline-final liraglutide	-2.523	0.590	0	-0.127	0.000
Baseline-final exenatide	-5.933	0.581	0	-0.240	0.000
GLP-1 RA compared to control	-6.759	0.085	0	-0.398	0.000
Baseline-final SGLT2i	0.621	0.816	0	-0.048	0.000
Baseline-final SGLT2i (RCTs)	5.190	0.629	0	-0.043	0.000

RCT: randomized clinical trial; T2DM: type 2 diabetes mellitus; GLP-1 RA: glucagon-like peptide-1 receptor agonists; SGLT2i: sodium-glucose cotransporter 2 inhibitors.

## Data Availability

Data is available on request from the corresponding author.

## Supplementary Materials

Supplementary Materials Table S1: search strategy of GLP-1 RA and SGLT2i. Table S2: quality assessment table for randomized clinical trial studies, cohort studies, cross-sectional studies, and nonrandomized studies based on JBI Critical Appraisal. Figure S1: GLP-1RA and IMT (sensitivity analysis on T2DM patients). Figure S2: liraglutide and exenatide effects on IMT. Figure S3: empagliflozin, tofogliflozin, and ipragliflozin effects on IMT. Figure S4: all analysis funnel plots (baseline-final comparison of GLP-1 RA (RCTs), baseline-final comparison of GLP-1 RA (T2DM), and baseline-final comparison of SGLT2i (RCTs)).
