# Supplementary material for: Effects of Glucagon-Like Peptide-1 Receptor Agonists and Sodium-Glucose Cotransporter 2 Inhibitors on Intima-Media Thickness: Systematic Review and Meta-Analysis
Source: J Diabetes Res. 2024 Mar 18;2024:3212795. doi: 10.1155/2024/3212795 (PMC10963118; doi:10.1155/2024/3212795)
Supplement: Supplementary Materials — Table S1: search strategy of GLP-1 RA and SGLT2i. Table S2: quality assessment table for randomized clinical trial studies, cohort studies, cross-sectional studies, and nonrandomized studies based on JBI Critical Appraisal. Figure S1: GLP-1RA and IMT (sensitivity analysis on T2DM patients). Figure S2: liraglutide and exenatide effects on IMT. Figure S3: empagliflozin, tofogliflozin, and ipragliflozin effects on IMT. Figure S4: all analysis funnel plots (baseline-final comparison of GLP-1 RA (RCTs), baseline-final comparison of GLP-1 RA (T2DM), and baseline-final comparison of SGLT2i (RCTs)). [file 3212795.f1.docx]

**Tables**

|  | **Table S1A: Search strategy of GLP-1 RA** | |
| --- | --- | --- |
|  | **Pubmed (September 9, 2023)** | |
| **Results** | **Query** | **Search** |
| 84,182 | (“intima media thickness*”[title/abstract] OR “intima-media thickness*”[title/abstract] OR IMT [title/abstract] OR CMIT[title/abstract] OR "carotid Atherosclero*"[title/abstract] OR "arterial Atherosclero*"[title/abstract] OR "artery Atherosclero*"[title/abstract] OR "artery Atherogene*"[title/abstract] OR "carotid Atherogene*" [title/abstract] OR "arterial Atherogene*" [title/abstract] OR “intima thickness” [title/abstract] OR “media thickness” [title/abstract] OR “thickness of intima” [title/abstract] OR “thickness of media” [title/abstract] OR “thickness of tunica” [title/abstract] OR "arterial steno*"[title/abstract] OR "artery steno*"[title/abstract] OR "carotid steno*"[title/abstract] OR "tunica intima*"[title/abstract] OR "carotid plaque*" OR "Arterial thickness"[title/abstract] OR "Arterial wall thickness"[title/abstract] OR " intima media complex” [title/abstract] OR "intimal medial complex"[title/abstract] OR “intimal media thickening” [title/abstract] OR “intima medial thickness” [title/abstract] OR “intima medial thickening” [title/abstract] OR “intima media complex”[title/abstract] OR “intimal medial complex”[title/abstract] OR “intimal media complex”[title/abstract] OR “intimamedia thickness”[title/abstract] OR “wall thickness”[title/abstract] OR “wall thickening”[title/abstract] OR “arterial thickness”[title/abstract] OR “artery thickness”[title/abstract] OR “artery wall thickness”[title/abstract] OR “arterial wall thickness”[title/abstract] ) | **#1** (IMT) |
| 22,743 | (“GLP-1”[title/abstract] OR “glucagon-like peptide-1”[title/abstract] OR “glucagon like peptide-1”[title/abstract] OR “glucagon-like peptide1”[title/abstract] OR “glucagon-like peptide 1”[title/abstract] OR “glucagon like peptide 1”[title/abstract] OR “incretin base*"[title/abstract] OR “incretin-base*"[title/abstract] OR “exenatide”[title/abstract] OR “liraglutide”[title/abstract] OR “dulaglutide”[title/abstract] OR “lixisenatide”[title/abstract] OR “semaglutide”[title/abstract] OR "albiglutide"[title/abstract] OR "taspoglutide"[title/abstract]) | **#2** (GLP-1 RA) |
| **64** | **#1 AND #2** | **Final** |
|  | **Scopus (September 8, 2023)** | |
| 192,471 | TITLE-ABS-KEY(“intima media thickness*” OR “intima-media thickness*” OR IMT OR CMIT OR "carotid Atherosclero*" OR "arterial Atherosclero*" OR "artery Atherosclero*" OR "artery Atherogene*" OR "carotid Atherogene*" OR "arterial Atherogene*" OR “intima thickness” OR “media thickness” OR “thickness of intima” OR “thickness of media” OR “thickness of tunica” OR "arterial steno*" OR "artery steno*" OR "carotid steno*" OR "tunica intima*" OR "carotid plaque*" OR "Arterial thickness" OR "Arterial wall thickness" OR " intima media complex” OR "intimal medial complex" OR “intimal media thickening” OR “intima medial thickness” OR “intima medial thickening” OR “intima media complex” OR “intimal medial complex” OR “intimal media complex” OR “intimamedia thickness” OR “wall thickness” OR “wall thickening” OR “arterial thickness” OR “artery thickness” OR “artery wall thickness” OR “arterial wall thickness” ) | **#1** (IMT) |
| 39,646 | TITLE-ABS-KEY(“GLP-1” OR “glucagon-like peptide-1” OR “glucagon like peptide-1” OR “glucagon-like peptide1” OR “glucagon-like peptide 1” OR “glucagon like peptide 1” OR “incretin base*" OR “incretin-base*" OR “exenatide” OR “liraglutide” OR “dulaglutide” OR “lixisenatide” OR “semaglutide” OR "albiglutide" OR "taspoglutide") | **#2** (GLP-1 RA) |
| **510** | **#1 AND #2** | **Final** |
|  | **Embase (September 8, 2023)** | |
| 130,493 | (“intima media thickness*”:ab,ti OR “intima-media thickness*”:ab,ti OR "IMT":ab,ti OR "CMIT":ab,ti OR "carotid Atherosclero*":ab,ti OR "arterial Atherosclero*":ab,ti OR "artery Atherosclero*":ab,ti OR "artery Atherogene*":ab,ti OR "carotid Atherogene*":ab,ti OR "arterial Atherogene*":ab,ti OR “intima thickness”:ab,ti OR “media thickness”:ab,ti OR “thickness of intima”:ab,ti OR “thickness of media”:ab,ti OR “thickness of tunica”:ab,ti OR "arterial steno*":ab,ti OR "artery steno*":ab,ti OR "carotid steno*":ab,ti OR "tunica intima*":ab,ti OR "carotid plaque*":ab,ti OR "Arterial thickness":ab,ti OR "Arterial wall thickness":ab,ti OR " intima media complex”:ab,ti OR "intimal medial complex":ab,ti OR “intimal media thickening”:ab,ti OR “intima medial thickness”:ab,ti OR “intima medial thickening”:ab,ti OR “intima media complex”:ab,ti OR “intimal medial complex”:ab,ti OR “intimal media complex”:ab,ti OR “intimamedia thickness”:ab,ti OR “wall thickness”:ab,ti OR “wall thickening”:ab,ti OR “arterial thickness”:ab,ti OR “artery thickness”:ab,ti OR “artery wall thickness”:ab,ti OR “arterial wall thickness”:ab,ti) | **#1** (IMT) |
| 36,657 | (“GLP-1”:ab,ti OR “glucagon-like peptide-1”:ab,ti OR “glucagon like peptide-1”:ab,ti OR “glucagon-like peptide1”:ab,ti OR “glucagon-like peptide 1”:ab,ti OR “glucagon like peptide 1”:ab,ti OR “incretin base*":ab,ti OR “incretin-base*":ab,ti OR “exenatide”:ab,ti OR “liraglutide”:ab,ti OR “dulaglutide”:ab,ti OR “lixisenatide”:ab,ti OR “semaglutide”:ab,ti OR "albiglutide":ab,ti OR "taspoglutide":ab,ti) | **#2** (GLP-1 RA) |
| **125** | **#1 AND #2** | **Final** |
|  | **Web Of Science (September 9, 2023)** | |
| 94,394 | (TI=(“intima media thickness*” OR “intima-media thickness*” OR IMT OR CMIT OR "carotid Atherosclero*" OR "arterial Atherosclero*" OR "artery Atherosclero*" OR "artery Atherogene*" OR "carotid Atherogene*" OR "arterial Atherogene*" OR “intima thickness” OR “media thickness” OR “thickness of intima” OR “thickness of media” OR “thickness of tunica” OR "arterial steno*" OR "artery steno*" OR "carotid steno*" OR "tunica intima*" OR "carotid plaque*" OR "Arterial thickness" OR "Arterial wall thickness" OR " intima media complex” OR "intimal medial complex" OR “intimal media thickening” OR “intima medial thickness” OR “intima medial thickening” OR “intima media complex” OR “intimal medial complex” OR “intimal media complex” OR “intimamedia thickness” OR “wall thickness” OR “wall thickening” OR “arterial thickness” OR “artery thickness” OR “artery wall thickness” OR “arterial wall thickness” ) OR AB=(“intima media thickness*” OR “intima-media thickness*” OR IMT OR CMIT OR "carotid Atherosclero*" OR "arterial Atherosclero*" OR "artery Atherosclero*" OR "artery Atherogene*" OR "carotid Atherogene*" OR "arterial Atherogene*" OR “intima thickness” OR “media thickness” OR “thickness of intima” OR “thickness of media” OR “thickness of tunica” OR "arterial steno*" OR "artery steno*" OR "carotid steno*" OR "tunica intima*" OR "carotid plaque*" OR "Arterial thickness" OR "Arterial wall thickness" OR " intima media complex” OR "intimal medial complex" OR “intimal media thickening” OR “intima medial thickness” OR “intima medial thickening” OR “intima media complex” OR “intimal medial complex” OR “intimal media complex” OR “intimamedia thickness” OR “wall thickness” OR “wall thickening” OR “arterial thickness” OR “artery thickness” OR “artery wall thickness” OR “arterial wall thickness” )) | **#1** (IMT) |
| 27,982 | ((TI=(“GLP-1” OR “glucagon-like peptide-1” OR “glucagon like peptide-1” OR “glucagon-like peptide1” OR “glucagon-like peptide 1” OR “glucagon like peptide 1” OR “incretin base*" OR “incretin-base*" OR “exenatide” OR “liraglutide” OR “dulaglutide” OR “lixisenatide” OR “semaglutide” OR "albiglutide" OR "taspoglutide")) OR AB=(“GLP-1” OR “glucagon-like peptide-1” OR “glucagon like peptide-1” OR “glucagon-like peptide1” OR “glucagon-like peptide 1” OR “glucagon like peptide 1” OR “incretin base*" OR “incretin-base*" OR “exenatide” OR “liraglutide” OR “dulaglutide” OR “lixisenatide” OR “semaglutide” OR "albiglutide" OR "taspoglutide")) | **#2** (GLP-1 RA) |
| **75** | **#1 AND #2** | **Final** |
|  | **Table S1B: Search strategy of SGLT2i** | |
|  | **Pubmed (September 9, 2023)** | |
| **Results** | **Query** | **Search** |
| 84,182 | (“intima media thickness*”[title/abstract] OR “intima-media thickness*”[title/abstract] OR IMT [title/abstract] OR CMIT[title/abstract] OR "carotid Atherosclero*"[title/abstract] OR "arterial Atherosclero*"[title/abstract] OR "artery Atherosclero*"[title/abstract] OR "artery Atherogene*"[title/abstract] OR "carotid Atherogene*" [title/abstract] OR "arterial Atherogene*" [title/abstract] OR “intima thickness” [title/abstract] OR “media thickness” [title/abstract] OR “thickness of intima” [title/abstract] OR “thickness of media” [title/abstract] OR “thickness of tunica” [title/abstract] OR "arterial steno*"[title/abstract] OR "artery steno*"[title/abstract] OR "carotid steno*"[title/abstract] OR "tunica intima*"[title/abstract] OR "carotid plaque*" OR "Arterial thickness"[title/abstract] OR "Arterial wall thickness"[title/abstract] OR " intima media complex” [title/abstract] OR "intimal medial complex"[title/abstract] OR “intimal media thickening” [title/abstract] OR “intima medial thickness” [title/abstract] OR “intima medial thickening” [title/abstract] OR “intima media complex”[title/abstract] OR “intimal medial complex”[title/abstract] OR “intimal media complex”[title/abstract] OR “intimamedia thickness”[title/abstract] OR “wall thickness”[title/abstract] OR “wall thickening”[title/abstract] OR “arterial thickness”[title/abstract] OR “artery thickness”[title/abstract] OR “artery wall thickness”[title/abstract] OR “arterial wall thickness”[title/abstract] ) | **#1** (IMT) |
| 12,152 | ("sodium-glucose cotransporter 2 inhibitors"[title/abstract] OR " sodium-glucose transporter 2 inhibitors"[title/abstract] OR SGLT2i[title/abstract] OR "SGLT 2i"[title/abstract] OR SGLT2Is[title/abstract] OR "SGLT 2is"[title/abstract] OR atigliflozin [title/abstract] OR AVE2268 [title/abstract] OR Dapaglifozin [title/abstract] OR PF04971729[title/abstract] OR "BMS 512148"[title/abstract] OR bexagliflozin [title/abstract] OR egt0001442 [title/abstract] OR egt0001474 [title/abstract] OR canagliflozin[title/abstract] OR invokana[title/abstract] OR " JNJ 28431754"[title/abstract] OR dapagliflozin[title/abstract] OR farxiga[title/abstract] OR Licogliflozin [title/abstract] OR egt0001474[title/abstract] OR empagliflozin[title/abstract] OR Topogliflozin [title/abstract] OR jardiance[title/abstract] OR "BI 10773"[title/abstract] OR BI10773 [title/abstract] OR ertugliflozin[title/abstract] OR ipragliflozin[title/abstract] OR suglat[title/abstract] OR asp1941[title/abstract] OR isis-sglt2rx[title/abstract] OR LX4211[title/abstract] OR luseogliflozin[title/abstract] OR lusefi[title/abstract] OR TS071[title/abstract] OR remogliflozin[title/abstract] OR bhv091009[title/abstract] OR sergliflozin[title/abstract] OR shr3824[title/abstract] OR sotagliflozin[title/abstract] OR "sodium-glucose cotransporter 2 inhibitor"[title/abstract] OR tofogliflozin[title/abstract] OR apleway[title/abstract] OR deberza[title/abstract] OR CSG452[title/abstract] OR (((((Na+/glucose[title/abstract] OR "sodium glucose"[title/abstract] OR "sodium dependent glucose"[title/abstract]) AND (transporter[title/abstract] OR cotransporter[title/abstract] OR "co transporter"[title/abstract])) OR ((SGLT2[title/abstract] OR SGLT 2[title/abstract]))) AND (inhibitor*[title/abstract)))) | **#2** (SGLT2i) |
| **36** | **#1 AND #2** | **Final** |
|  | **Scopus (September 5, 2023)** | |
| 192,471 | TITLE-ABS-KEY(“intima media thickness*” OR “intima-media thickness*” OR IMT OR CMIT OR "carotid Atherosclero*" OR "arterial Atherosclero*" OR "artery Atherosclero*" OR "artery Atherogene*" OR "carotid Atherogene*" OR "arterial Atherogene*" OR “intima thickness” OR “media thickness” OR “thickness of intima” OR “thickness of media” OR “thickness of tunica” OR "arterial steno*" OR "artery steno*" OR "carotid steno*" OR "tunica intima*" OR "carotid plaque*" OR "Arterial thickness" OR "Arterial wall thickness" OR " intima media complex” OR "intimal medial complex" OR “intimal media thickening” OR “intima medial thickness” OR “intima medial thickening” OR “intima media complex” OR “intimal medial complex” OR “intimal media complex” OR “intimamedia thickness” OR “wall thickness” OR “wall thickening” OR “arterial thickness” OR “artery thickness” OR “artery wall thickness” OR “arterial wall thickness” ) | **#1** (IMT) |
| 7,062 | TITLE-ABS-KEY("sodium-glucose cotransporter 2 inhibitors" OR "sodium-glucose transporter 2 inhibitors" OR SGLT2i OR "SGLT 2i" OR SGLT2Is OR "SGLT 2is" OR atigliflozin or AVE2268 OR Dapaglifozin or PF04971729 or "BMS 512148" OR bexagliflozin OR egt0001442 OR egt0001474 OR canagliflozin OR invokana or "JNJ 28431754" OR dapagliflozin OR farxiga OR egt0001474 OR empagliflozin OR jardiance or "BI 10773" OR BI10773 OR ertugliflozin OR ipragliflozin OR suglat OR asp1941 OR isis-sglt2rx OR LX4211 OR luseogliflozin OR "lusefior TS071" OR "sodium-glucose cotransporter 2 inhibitor" or remogliflozin OR bhv091009 OR sergliflozin OR shr3824 OR sotagliflozin OR tofogliflozin OR apleway OR deberzaor CSG452 OR ( ( na+/glucose OR "sodium glucose" OR "sodium dependent glucose" ) AND ( transporter OR cotransporter OR "co transporter" ) ) OR ((SGLT2 OR SGLT 2) AND inhibitor*)) | **#2** (SGLT2i) |
| **124** | **#1 AND #2** | **Final** |
|  | **Embase (September 5, 2023)** | |
| 130,493 | (“intima media thickness*”:ab,ti OR “intima-media thickness*”:ab,ti OR "IMT":ab,ti OR "CMIT":ab,ti OR "carotid Atherosclero*":ab,ti OR "arterial Atherosclero*":ab,ti OR "artery Atherosclero*":ab,ti OR "artery Atherogene*":ab,ti OR "carotid Atherogene*":ab,ti OR "arterial Atherogene*":ab,ti OR “intima thickness”:ab,ti OR “media thickness”:ab,ti OR “thickness of intima”:ab,ti OR “thickness of media”:ab,ti OR “thickness of tunica”:ab,ti OR "arterial steno*":ab,ti OR "artery steno*":ab,ti OR "carotid steno*":ab,ti OR "tunica intima*":ab,ti OR "carotid plaque*":ab,ti OR "Arterial thickness":ab,ti OR "Arterial wall thickness":ab,ti OR " intima media complex”:ab,ti OR "intimal medial complex":ab,ti OR “intimal media thickening”:ab,ti OR “intima medial thickness”:ab,ti OR “intima medial thickening”:ab,ti OR “intima media complex”:ab,ti OR “intimal medial complex”:ab,ti OR “intimal media complex”:ab,ti OR “intimamedia thickness”:ab,ti OR “wall thickness”:ab,ti OR “wall thickening”:ab,ti OR “arterial thickness”:ab,ti OR “artery thickness”:ab,ti OR “artery wall thickness”:ab,ti OR “arterial wall thickness”:ab,ti) | **#1** (IMT) |
| 21,195 | ("sodium-glucose cotransporter 2 inhibitors":ab,ti OR "sodium-glucose transporter 2 inhibitors":ab,ti OR "sglt2i":ab,ti OR "sglt 2i":ab,ti OR "sglt2is":ab,ti OR "sglt 2is":ab,ti OR "atigliflozin":ab,ti OR "ave2268":ab,ti OR "dapaglifozin":ab,ti OR "pf04971729":ab,ti OR "bms 512148":ab,ti OR "bexagliflozin":ab,ti OR "egt0001442":ab,ti OR "canagliflozin":ab,ti OR "invokana":ab,ti OR "jnj 28431754":ab,ti OR "dapagliflozin":ab,ti OR "farxiga":ab,ti OR  "egt0001474":ab,ti OR "empagliflozin":ab,ti OR "jardiance":ab,ti OR "bi 10773":ab,ti OR "bi10773":ab,ti OR "ertugliflozin":ab,ti OR "ipragliflozin":ab,ti OR "suglat":ab,ti OR "asp1941":ab,ti OR "isis sglt2rx":ab,ti OR "lx4211":ab,ti OR "luseogliflozin":ab,ti OR "lusefior ts071":ab,ti OR "remogliflozin":ab,ti OR "bhv091009":ab,ti OR "sergliflozin":ab,ti OR "sodium-glucose cotransporter 2 inhibitor":ti,ab or "shr3824":ab,ti OR "sotagliflozin":ab,ti OR "tofogliflozin":ab,ti OR "apleway":ab,ti OR "deberzaor csg452":ab,ti OR (("na+/glucose":ab,ti OR "sodium glucose":ab,ti OR "sodium dependent glucose":ab,ti) AND ("transporter":ab,ti OR "cotransporter":ab,ti OR "co transporter":ab,ti)) OR (("sglt2":ab,ti OR "sglt 2":ab,ti) AND "inhibitor*":ab,ti)) | **#2** (SGLT2i) |
| **67** | **#1 AND #2** | **Final** |
|  | **Web Of Science (September 9, 2023)** | |
| 94,394 | (TI=(“intima media thickness*” OR “intima-media thickness*” OR IMT OR CMIT OR "carotid Atherosclero*" OR "arterial Atherosclero*" OR "artery Atherosclero*" OR "artery Atherogene*" OR "carotid Atherogene*" OR "arterial Atherogene*" OR “intima thickness” OR “media thickness” OR “thickness of intima” OR “thickness of media” OR “thickness of tunica” OR "arterial steno*" OR "artery steno*" OR "carotid steno*" OR "tunica intima*" OR "carotid plaque*" OR "Arterial thickness" OR "Arterial wall thickness" OR " intima media complex” OR "intimal medial complex" OR “intimal media thickening” OR “intima medial thickness” OR “intima medial thickening” OR “intima media complex” OR “intimal medial complex” OR “intimal media complex” OR “intimamedia thickness” OR “wall thickness” OR “wall thickening” OR “arterial thickness” OR “artery thickness” OR “artery wall thickness” OR “arterial wall thickness” ) OR AB=(“intima media thickness*” OR “intima-media thickness*” OR IMT OR CMIT OR "carotid Atherosclero*" OR "arterial Atherosclero*" OR "artery Atherosclero*" OR "artery Atherogene*" OR "carotid Atherogene*" OR "arterial Atherogene*" OR “intima thickness” OR “media thickness” OR “thickness of intima” OR “thickness of media” OR “thickness of tunica” OR "arterial steno*" OR "artery steno*" OR "carotid steno*" OR "tunica intima*" OR "carotid plaque*" OR "Arterial thickness" OR "Arterial wall thickness" OR " intima media complex” OR "intimal medial complex" OR “intimal media thickening” OR “intima medial thickness” OR “intima medial thickening” OR “intima media complex” OR “intimal medial complex” OR “intimal media complex” OR “intimamedia thickness” OR “wall thickness” OR “wall thickening” OR “arterial thickness” OR “artery thickness” OR “artery wall thickness” OR “arterial wall thickness” )) | **#1** (IMT) |
| 16,595 | ((TI=("sodium-glucose cotransporter 2 inhibitors" OR "sodium-glucose transporter 2 inhibitors" OR SGLT2i OR "SGLT 2i" OR SGLT2Is OR "SGLT 2is" OR atigliflozin or AVE2268 OR Dapaglifozin or PF04971729 or "BMS 512148" OR bexagliflozin OR egt0001442 OR egt0001474 OR canagliflozin OR invokana or "JNJ 28431754" OR dapagliflozin OR farxiga OR egt0001474 OR empagliflozin OR jardiance or "BI 10773" OR BI10773 OR "sodium-glucose cotransporter 2 inhibitor" or ertugliflozin OR ipragliflozin OR suglat OR asp1941 OR isis-sglt2rx OR LX4211 OR luseogliflozin OR "lusefior TS071" OR remogliflozin OR bhv091009 OR sergliflozin OR shr3824 OR sotagliflozin OR tofogliflozin OR apleway OR deberzaor CSG452 OR ( ( na+/glucose OR "sodium glucose" OR "sodium dependent glucose" ) AND ( transporter OR cotransporter OR "co transporter" ) ) OR ((SGLT2 OR SGLT 2) AND inhibitor*)) OR AB=("sodium-glucose cotransporter 2 inhibitors" OR "sodium-glucose transporter 2 inhibitors" OR SGLT2i OR "SGLT 2i" OR SGLT2Is OR "SGLT 2is" OR atigliflozin or AVE2268 OR Dapaglifozin or PF04971729 or "BMS 512148" OR bexagliflozin OR egt0001442 OR egt0001474 OR canagliflozin OR invokana or "JNJ 28431754" OR dapagliflozin OR farxiga OR egt0001474 OR empagliflozin OR jardiance or "BI 10773" OR BI10773 OR "sodium-glucose cotransporter 2 inhibitor" or ertugliflozin OR ipragliflozin OR suglat OR asp1941 OR isis-sglt2rx OR LX4211 OR luseogliflozin OR "lusefior TS071" OR remogliflozin OR bhv091009 OR sergliflozin OR shr3824 OR sotagliflozin OR tofogliflozin OR apleway OR deberzaor CSG452 OR ( ( na+/glucose OR "sodium glucose" OR "sodium dependent glucose" ) AND ( transporter OR cotransporter OR "co transporter" ) ) OR ((SGLT2 OR SGLT 2) AND inhibitor*)))) | **#2** (SGLT2i) |
| **40** | **#1 AND #2** | **Final** |

Table S2A. Quality assessment table for randomized clinical trial studies based on JBI Critical Appraisal.

| Study | 1. Was true randomization used for assignment of participants to treatment groups? | 2. Was allocation to treatment groups concealed? | 3. Were treatment groups similar at the baseline? | 4. Were participants blind to treatment assignment? | 5. Were those delivering treatment blind to treatment assignment? | 6. Were outcomes assessors blind to treatment assignment? | 7. Were treatment groups treated identically other than the intervention of interest? | 8. Was follow up complete and if not, were differences between groups in terms of their follow up adequately described and analyzed? | 9. Were participants analyzed in the groups to which they were randomized? | 10. Were outcomes measured in the same way for treatment groups? | 11. Were outcomes measured in a reliable way? | 12. Was appropriate statistical analysis used? | 13. Was the trial design appropriate, and any deviations from the standard RCT design (individual randomization, parallel groups) accounted for in the conduct and analysis of the trial? | Final score |
| --- | --- | --- | --- | --- | --- | --- | --- | --- | --- | --- | --- | --- | --- | --- |
| **Dejgaard, 2016** | U | U | Y | Y | Y | N | Y | Y | Y | Y | U | U | U | 7 |
| **Zhang, 2015** | Y | N | Y | N | N | N | N | Y | Y | Y | U | Y | Y | 7 |
| **Meng, 2021** | Y | N | Y | N | N | N | N | Y | Y | Y | Y | Y | Y | 8 |
| **Ripa, 2017** | Y | Y | Y | Y | Y | N | Y | Y | Y | Y | Y | Y | Y | 12 |
| **Sun, 2009** | Y | Y | Y | Y | Y | N | Y | Y | Y | Y | Y | Y | Y | 12 |
| **Kang, 2017** | Y | N | Y | N | N | N | N | Y | Y | Y | Y | Y | Y | 8 |
| **Katakami, 2016** | Y | N | Y | N | N | N | N | Y | Y | Y | Y | Y | Y | 8 |
| **Murakami, 2014** | U | N | Y | N | N | N | N | Y | Y | Y | Y | U | U | 5 |
| **Tanaka, 2015** | Y | N | Y | N | N | N | N | Y | Y | Y | U | Y | Y | 7 |

Y: Yes, N: No, U: Unclear

Table S2B. Quality assessment table for cross-sectional studies based on JBI Critical Appraisal.

| Study | 1 Were the criteria for inclusion in the sample clearly defined? | 2 Were the study subjects and the setting described in detail? | 3 Was the exposure measured in a valid and reliable way? | 4 Were objective, standard criteria used for measurement of the condition? | 5 Were confounding factors identified? | 6 Were strategies to deal with confounding factors stated? | 7 Were the outcomes measured in a valid and reliable way? | 8 Was appropriate statistical analysis used? | Final score |
| --- | --- | --- | --- | --- | --- | --- | --- | --- | --- |
| **Luna-Marco, 2023** | Y | Y | Y | Y | Y | Y | Y | Y | 8 |
| **Kourtidou, 2021** | Y | Y | Y | Y | Y | Y | Y | Y | 8 |

Y: Yes

Table S2C. Quality assessment table for quasi-experimental studies based on JBI Critical Appraisal.

| Study | 1. Is it clear in the study what is the ‘cause’ and what is the ‘effect’ (i.e. there is no confusion about which variable comes first)? | 2. Were the participants included in any comparisons similar? | 3. Were the participants included in any comparisons receiving similar treatment/care, other than the exposure or intervention of interest? | 4. Was there a control group? | 5. Were there multiple measurements of the outcome both pre and post the intervention/exposure? | 6. Was follow up complete and if not, were differences between groups in terms of their follow up adequately described and analyzed? | 7. Were the outcomes of participants included in any comparisons measured in the same way? | 8. Were outcomes measured in a reliable way? | 9. Was appropriate statistical analysis used? |  |
| --- | --- | --- | --- | --- | --- | --- | --- | --- | --- | --- |
| **Rizzo, 2012** | Y | NA | NA | N | Y | Y | Y | Y | Y | 6 |
| **Nikolic, 2012** | Y | NA | NA | N | Y | Y | Y | Y | Y | 6 |
| **Giglio, 2012** | Y | N | Y | Y | Y | Y | Y | Y | Y | 8 |
| **Patti, 2015** | Y | NA | NA | N | Y | Y | Y | Y | Y | 6 |
| **Hopkins, 2012** | Y | NA | NA | N | Y | Y | Y | Y | Y | 6 |
| **Kahal, 2015** | Y | N | Y | Y | Y | Y | Y | Y | Y | 8 |
| **Köseoğlu, 2015** | Y | NA | NA | N | Y | Y | Y | Y | Y | 6 |
| **Patti, 2013** | Y | NA | NA | N | Y | Y | Y | Y | Y | 6 |
| **Rizzo, 2012** | Y | NA | NA | N | Y | Y | Y | Y | Y | 6 |
| **Yoshida, 2018** | Y | Y | N | Y | Y | Y | Y | U | U | 4 |
| **Yoshida, 2012** | Y | Y | N | Y | Y | Y | Y | Y | Y | 6 |
| **Korzh, 2020** | Y | NA | NA | N | Y | Y | Y | U | U | 4 |
| **Lamaida, 2019** | Y | Y | Y | Y | Y | Y | Y | Y | Y | 9 |
| **Nomiyama, 2018** | Y | NA | NA | N | Y | Y | Y | Y | Y | 6 |
| **Sakai, 2015** | Y | Y | Y | Y | Y | Y | Y | U | Y | 8 |
| **Yamagishi, 2016** | Y | NA | NA | N | Y | Y | Y | U | U | 4 |

Y: Yes, N: No, U: Unclear, NA: Not applicable

Table S2D. Quality assessment table for cohort studies based on JBI Critical Appraisal.

| Study | 1. Were the two groups similar and recruited from the same population? | 2. Were the exposures measured similarly to assign people to both exposed and unexposed groups? | 3. Was the exposure measured in a valid and reliable way? | 4.Were confounding factors identified? | 5. Were strategies to deal with confounding factors stated? | 6. Were the groups/participants free of the outcome at the start of the study (or at the moment of exposure)? | 7. Were the outcomes measured in a valid and reliable way? | 8. Was the follow up time reported and sufficient to be long enough for outcomes to occur? | 9. Was follow up complete, and if not, were the reasons to loss to follow up described and explored? | 10. Were strategies to address incomplete follow up utilized? | 11. Was appropriate statistical analysis used? |  |
| --- | --- | --- | --- | --- | --- | --- | --- | --- | --- | --- | --- | --- |
| **Patti, 2019** | NA | Y | Y | Y | Y | Y | Y | Y | Y | NA | Y | 9 |
| **Irace, 2018** | Y | Y | Y | Y | Y | Y | Y | Y | Y | NA | Y | 10 |

Y: Yes, NA: Not applicable

**Figures**

Figure S1. GLP-1RA and IMT (sensitivity analysis on T2DM patients)


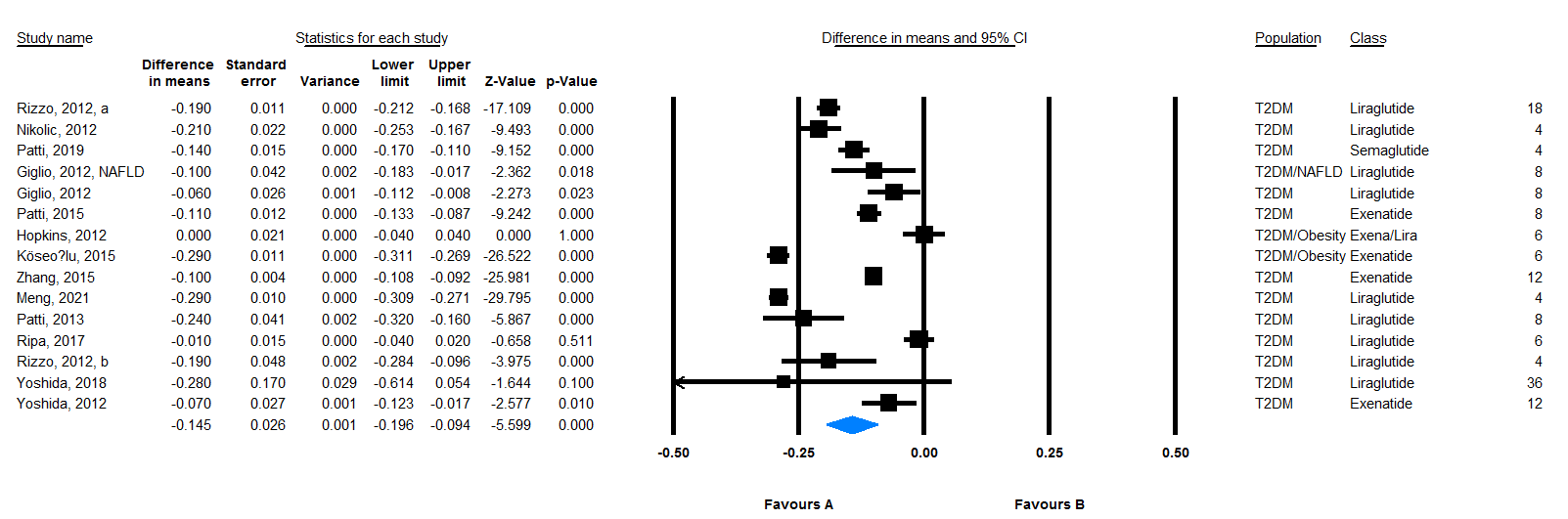

Figure S2. Liraglutide and exenatide effects on IMT.

Liraglutide:


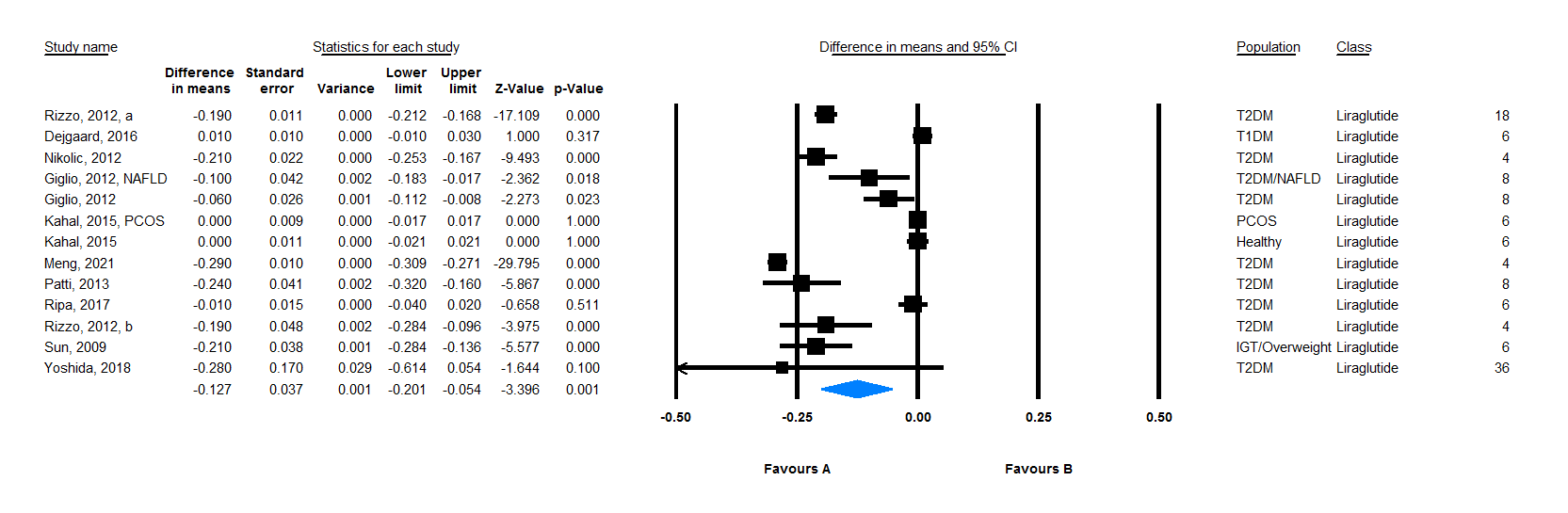


Exenatide:


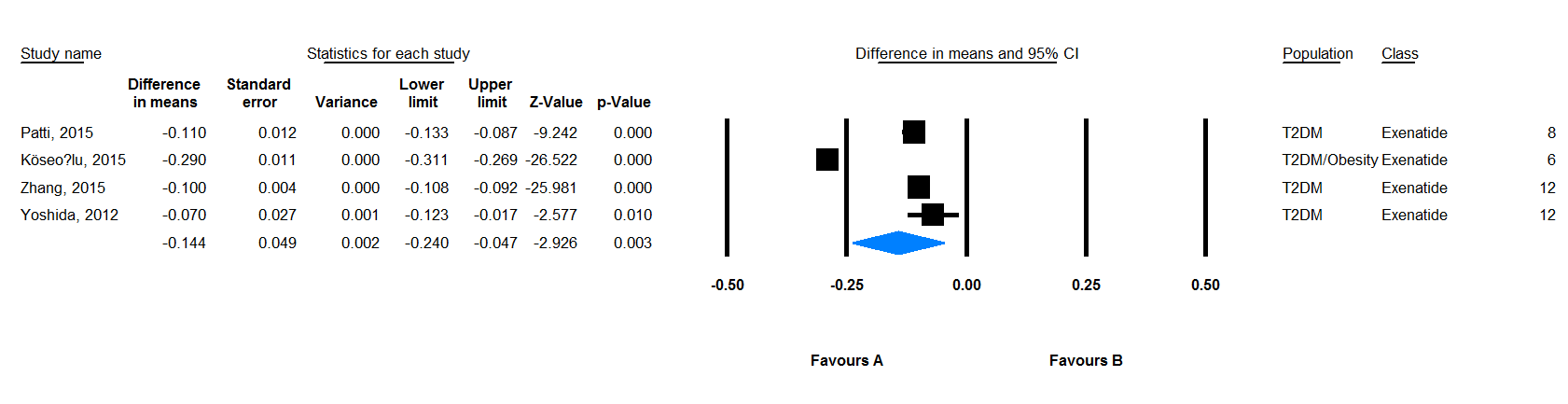

Figure S3. Empagliflozin, tofogliflozin, and ipragliflozin effects on IMT.

Empagliflozin:


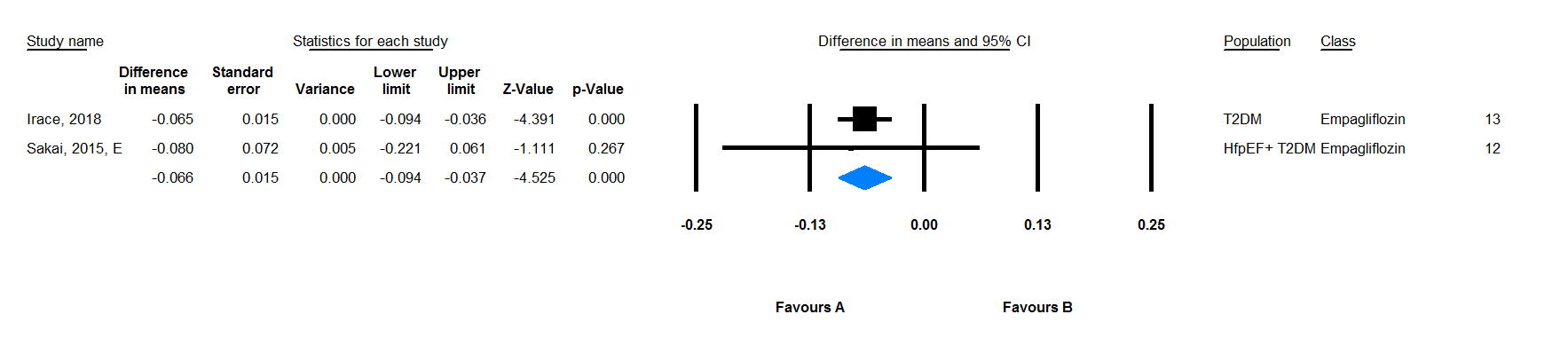


Tofogliflozin:


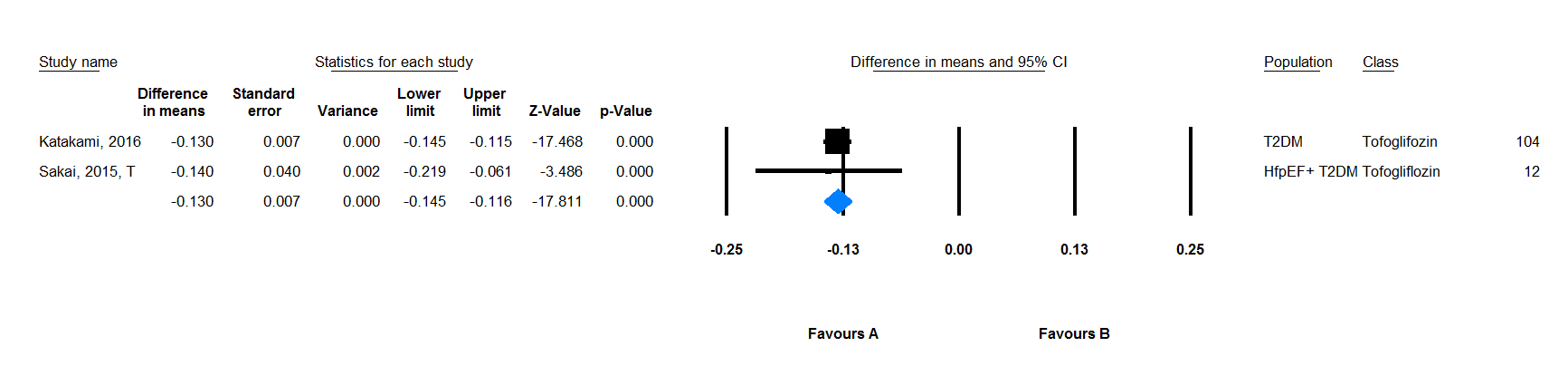


Ipragliflozin:


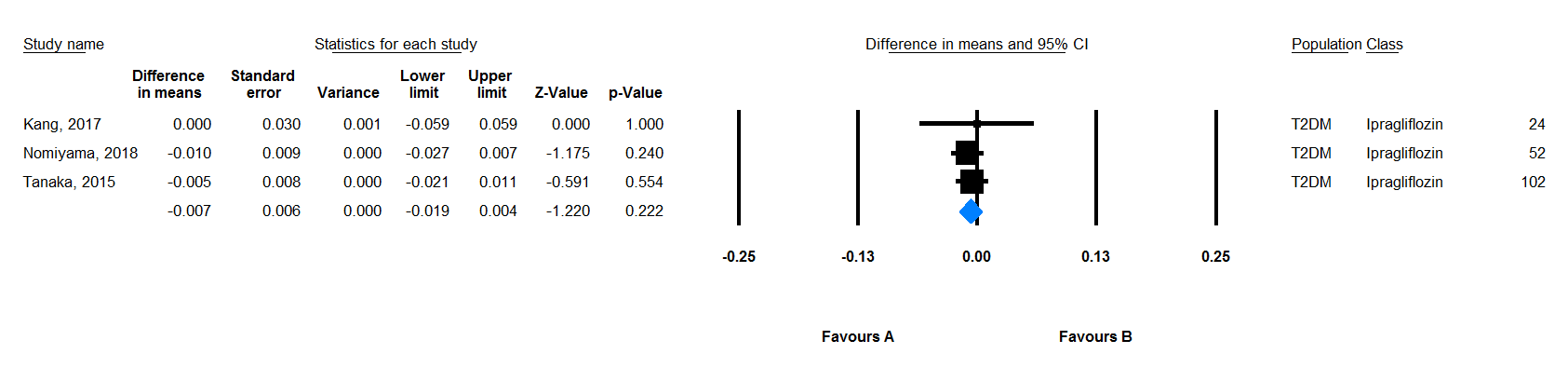


Figure S4. All analysis funnel plots.

Baseline-final comparision of GLP-1 RA (RCTs)

Baseline-final comparision of GLP-1 RA (T2DM)

Baseline-final comparision of SGLT2i (RCTs)
